# Pulmonary disease caused by *Mycobacterium meguroense*, a newly identified slow-growing mycobacterium, after umbilical cord blood transplantation: A case report from Japan and a mini-review of the literature

**DOI:** 10.1016/j.nmni.2026.101865

**Published:** 2026-10-08

**Authors:** Shin Iizuka, Mitsunori Yoshida, Taro Shinozaki, Yoshifumi Uwamino, Rika Inose, Yoshitaka Oyamada, Koichi Fukunaga, Takanori Asakura, Ho Namkoong

**Affiliations:** a Division of Pulmonary Medicine, Department of Medicine, Keio University School of Medicine, Tokyo, Japan; b Department of Mycobacteriology, Leprosy Research Center, National Institute of Infectious Diseases, Japan Institute for Health Security, Tokyo, Japan; c Department of Respiratory Medicine, National Hospital Organization Tokyo Medical Center, Tokyo, Japan; d Department of Laboratory Medicine, Keio University School of Medicine, Tokyo, Japan; e Clinical Laboratory, Keio University Hospital, Tokyo, Japan; f Department of Infectious Diseases, Keio University School of Medicine, Tokyo, Japan

**Keywords:** Bronchiectasis, Hematopoietic stem cell transplantation, Drug susceptibility testing, Phylogeny, Fluoroquinolones

## Abstract

We report pulmonary disease associated with an unclassified nontuberculous mycobacterium after umbilical cord blood transplantation. Genomic analyses assigned our isolates to the same species as feline strain MFM001, a species for which we propose the name *Mycobacterium meguroense*. Multidrug therapy was followed by clinical improvement and mycobacterial culture conversion.

## 1 Introduction

Nontuberculous mycobacterial pulmonary disease (NTM-PD) is increasingly recognized worldwide, with rising prevalence and substantial morbidity [1]. Multilocus sequence analysis and whole-genome sequencing (WGS) have revealed novel NTM species that are not identifiable by conventional methods. We report pulmonary disease caused by an unclassified NTM after umbilical cord blood transplantation (UCBT). The organism belongs to the species previously reported as strain MFM001, for which we propose the name *Mycobacterium meguroense*.

## 2 Case presentation

A 73-year-old woman with acute myeloid leukemia in remission after UCBT at age 67 was referred for evaluation of NTM infection. Comorbidities included myocardial infarction, diabetes mellitus, dyslipidemia, and hypertension. One year before referral, she developed cough and hemoptysis. Sputum mycobacterial cultures were positive from symptom onset, although the organism could not be identified at that time. *Pseudomonas aeruginosa* was not isolated initially and was first detected approximately 11 months after symptom onset, while mycobacterial cultures remained positive. Chest computed tomography revealed peripheral granular opacities with bronchiectasis, bronchial wall thickening, and mucus plugging in both lower lobes (Fig. S1). Acid-fast smears were also repeatedly positive. Two mycobacterial isolates (KO_240205 and KO_240413), obtained from sputum specimens collected on different dates, were not identified by MALDI-TOF MS, and sequencing of the 16S rRNA, *hsp65*, and *rpoB* genes showed no 100% BLAST match. Taken together, these findings fulfilled the clinical, radiological, and microbiological diagnostic criteria for NTM-PD [2]. Because respiratory symptoms and repeatedly positive mycobacterial cultures preceded the first isolation of *P. aeruginosa* by approximately 11 months, we considered the mycobacterium to have contributed to the pulmonary disease, although a role for *P. aeruginosa* could not be excluded. We performed WGS to clarify the taxonomic position of the isolates. Assembly statistics are provided in Table S1. Phylogenomic analysis incorporating 227 reference genomes, including *Mycobacterium* sp. strain MFM001 (Table S2), placed the clinical isolates in the same cluster as MFM001 (Fig. 1A). The two clinical isolates shared 100.00% average nucleotide identity (ANI). Each isolate showed 99.98% ANI and 99.8% digital DNA–DNA hybridization (dDDH; 95% confidence interval, 99.7–99.9%) with MFM001, supporting assignment of both to the same species. In contrast, their ANI and dDDH values with *M. branderi*, *M. celatum*, and *M. kyorinense* were 90.08–90.64% and 37.9–39.7%, respectively (Fig. 1B; Table S3). Broth microdilution with BrothMIC SGM yielded low clarithromycin minimum inhibitory concentrations (MICs) of 0.125 and 0.25 μg/mL (Table S4).

**Fig. 1:**
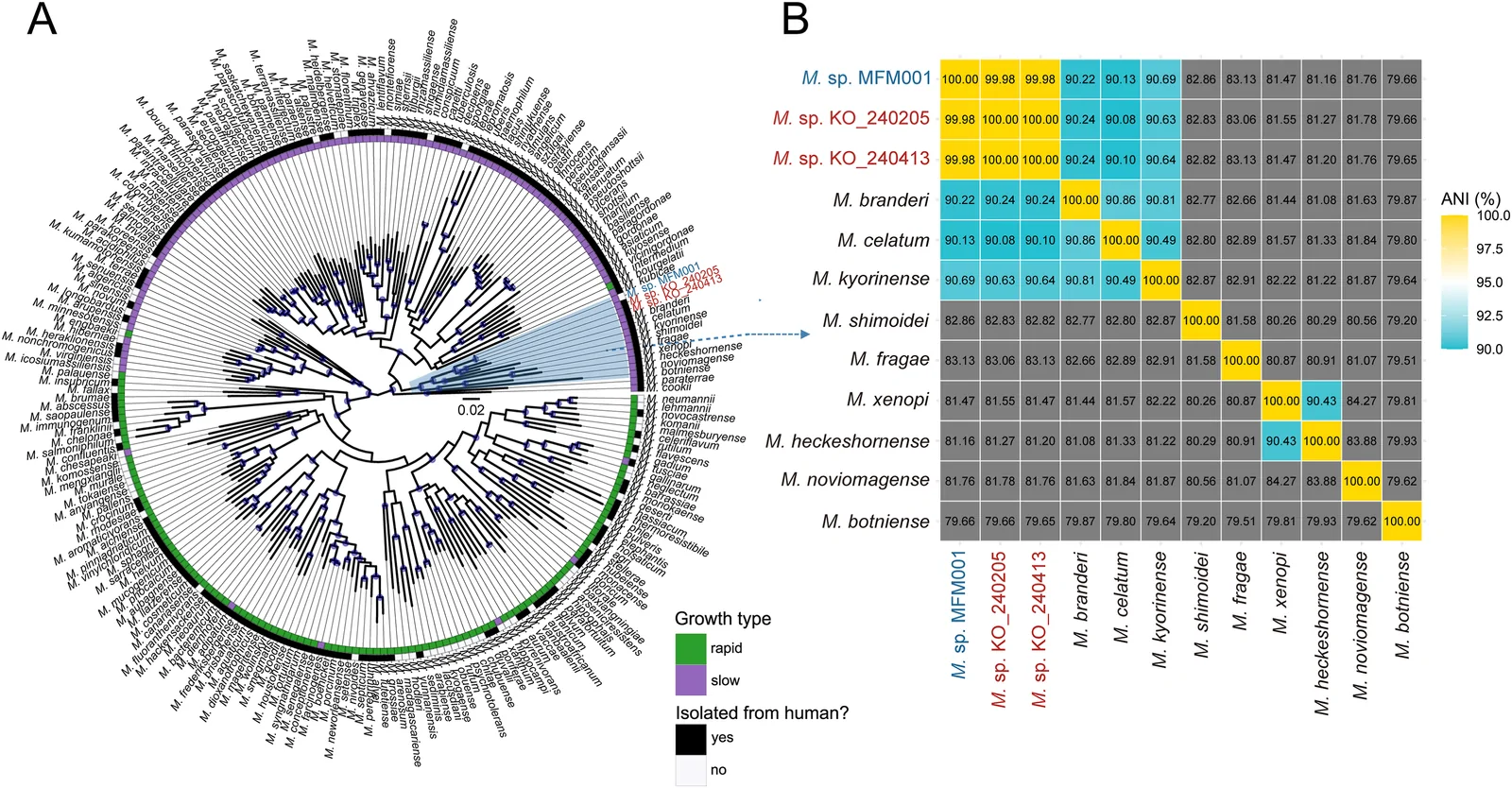
Phylogenomic placement and genomic relatedness of the clinical isolates. (A) Maximum-likelihood phylogenetic tree constructed from core-gene alignments of the two clinical isolates and 227 reference genomes, including *Mycobacterium* sp. strain MFM001. The clinical isolates and MFM001 are highlighted in red and blue, respectively. Colored rings indicate growth type and reported human isolation, as shown in the key. Branch lengths represent the estimated number of nucleotide substitutions per site; the scale bar indicates 0.02 substitutions per site. (B) Pairwise average nucleotide identity (ANI) heatmap for the clinical isolates, MFM001, and selected related taxa. The related taxa were selected based on their phylogenetic proximity to the clinical isolates in panel A. The clinical isolates share 100.00% ANI with each other and 99.98% ANI with MFM001. Corresponding ANI and digital DNA–DNA hybridization values for comparisons with MFM001 and the three closely related species are provided in Table S3.

Five months after referral, she began clarithromycin 800 mg/day, ethambutol 500 mg/day, and levofloxacin 500 mg/day, and clinical improvement followed. The last positive sputum cultures for both the mycobacterium and *P. aeruginosa* were obtained in the month treatment began, and neither organism was recovered thereafter. She has continued treatment for 1 year without relapse.

## 3 Discussion

Published clinical information on the species represented by *Mycobacterium* sp. strain MFM001 remains limited. Kayanuma et al. reported disseminated infection with granulomatous gastroenterocolitis in a cat receiving immunosuppressive treatment [3]. They also reported partial 16S rRNA gene sequence identity between MFM001 and strain B10-07.09.0206, isolated from a human pulmonary infection in Germany [3]. This partial-gene match suggests a relationship with an earlier human isolate, but species identity was not confirmed by whole-genome comparison.

At the genomic level, our analysis links the present clinical isolates to the previously reported feline strain. Both isolates showed 99.98% ANI and 99.8% dDDH with MFM001, supporting their assignment to the same species (Fig. 1; Table S3). Accordingly, we propose the name *M. meguroense* for this previously reported, unnamed species. The present case contributes genomic characterization and a detailed description of pulmonary disease after UCBT rather than reporting this organism for the first time. Together, the feline report and the present case suggest that this species can be associated with disease in different mammalian hosts, but its reservoir and routes of acquisition remain uncertain.

Reports of the closely related species *M. branderi*, *M. celatum*, and *M. kyorinense* provide additional clinical context, although they cannot establish an effective regimen for *M. meguroense*. Turvey et al. described *M. branderi* pulmonary disease that progressed despite treatment with clarithromycin, ethambutol, and moxifloxacin and ultimately required surgical resection [4]. Jun et al. reported successful treatment of *M. celatum* pulmonary disease with clarithromycin, ethambutol, and ciprofloxacin plus resection of a persistent cavity [5]. Nagao et al. described *M. kyorinense* pulmonary disease following cavitary tuberculosis, treated with clarithromycin, moxifloxacin, and an initial course of amikacin [6]. Across these reports of pulmonary disease caused by related slow-growing mycobacteria, multidrug regimens were used and treatment courses varied.

In the present case, the low clarithromycin MICs provided microbiological support for including a macrolide in the treatment regimen (Table S4). However, phylogenetic relatedness alone does not establish shared antimicrobial susceptibility or a similar clinical response. The improvement seen during clarithromycin, ethambutol, and levofloxacin treatment therefore remains a single clinical observation rather than evidence of a preferred regimen for this species. Further well-characterized cases are needed to define the clinical spectrum of this species, the relationship between MICs and treatment outcomes, and the optimal combination and duration of therapy.

In a patient after UCBT, this case provides genomic and clinical evidence of pulmonary disease associated with the species previously reported as strain MFM001, for which we propose the name *M. meguroense*. Accurate molecular identification and careful assessment of competing causes of pulmonary disease are essential when evaluating the clinical significance of rare or unclassified NTM.

## CRediT authorship contribution statement

**Shin Iizuka:** Investigation, Conceptualization, Data curation, Visualization, Writing – original draft. **Mitsunori Yoshida:** Investigation, Conceptualization, Data curation, Formal analysis, Methodology, Resources, Visualization, Writing – original draft. **Taro Shinozaki:** Investigation, Data curation, Writing – review & editing. **Yoshifumi Uwamino:** Investigation, Data curation, Supervision, Writing – review & editing. **Rika Inose:** Investigation, Data curation, Resources, Writing – review & editing. **Yoshitaka Oyamada:** Investigation, Data curation, Writing – review & editing. **Koichi Fukunaga:** Investigation, Supervision, Writing – review & editing. **Takanori Asakura:** Investigation, Conceptualization, Project administration, Supervision, Writing – review & editing. **Ho Namkoong:** Investigation, Conceptualization, Project administration, Supervision, Writing – review & editing.

## Funding sources

This study was supported by the Japan Agency for Medical Research and Development (AMED) under Grant Number JP26fk0108750; Japan Society for the Promotion of Science (JSPS) KAKENHI 25K02695.

## Declaration of competing interest

The authors declare that they have no known competing financial interests or personal relationships that could have appeared to influence the work reported in this paper.
