# Supplementary material for: Pulmonary disease caused by Mycobacterium meguroense, a newly identified slow-growing mycobacterium, after umbilical cord blood transplantation: A case report from Japan and a mini-review of the literature
Source: New Microbes New Infect. 2026 Oct 8;74:101865. doi: 10.1016/j.nmni.2026.101865 (PMC13716992; doi:10.1016/j.nmni.2026.101865)
Supplement: Supplementary file 1 [file mmc1.docx]

**Online Supplement**

**Pulmonary disease caused by *Mycobacterium meguroense*, a newly identified slow-growing mycobacterium, after umbilical cord blood transplantation: A case report from Japan and a mini-review of the literature**

Running title: *M. meguroense* pulmonary disease after UCBT

Shin Iizuka^1#^, Mitsunori Yoshida^2#^, Taro Shinozaki^3^, Yoshifumi Uwamino^4^, Rika Inose^5^, Yoshitaka Oyamada^3^, Koichi Fukunaga^1^, Takanori Asakura^1^*****, Ho Namkoong^6^*****

^1^Division of Pulmonary Medicine, Department of Medicine, Keio University School of Medicine, Tokyo, Japan

^2^Department of Mycobacteriology, Leprosy Research Center, National Institute of Infectious Diseases, Japan Institute for Health Security, Tokyo, Japan.

^3^Department of Respiratory Medicine, National Hospital Organization Tokyo Medical Center, Tokyo, Japan.

^4^Department of Laboratory Medicine, Keio University School of Medicine, Tokyo, Japan.

^5^Clinical Laboratory, Keio University Hospital, Tokyo, Japan.

^6^Department of Infectious Diseases, Keio University School of Medicine, Tokyo, Japan.

^#^These authors contributed equally to this work.

***Corresponding authors**

Takanori Asakura, MD, PhD

Division of Pulmonary Medicine, Department of Medicine, Keio University School of Medicine, 35 Shinanomachi, Shinjuku, Tokyo, Japan 160-8582

Email: [takanori.asakura@keio.jp](mailto:takanori.asakura@keio.jp)

Tel: +81-3-3353-1211

Ho Namkoong, MD, PhD, MPH

Department of Infectious Diseases, Keio University School of Medicine,

35 Shinanomachi, Shinjuku, Tokyo, Japan 160-8582

Email: [hounamugun@keio.jp](mailto:hounamugun@keio.jp)

Tel: +81-3-3353-1211

The authors have no conflicts of interest to disclose.

**Supplementary Methods**

**DNA sequencing and genomic analysis**

Genomic DNA was extracted from each isolate with a NucleoSpin Plant II kit (MACHEREY-NAGEL, Düren, Germany) in accordance with the manufacturer’s instructions. The extracted DNA was then used for NEBNext Ultra II DNA library construction and genome sequencing on an Illumina NovaSeq 6000. Raw read data generated in this study were deposited in the DNA Data Bank of Japan (DDBJ) and are mirrored at the National Center for Biotechnology Information (NCBI) under BioProject accession number PRJDB40215.

The Illumina read data for each isolate were assembled *de novo* using the Shovill pipeline with the “--trim” option (https://github.com/tseemann/shovill). After assembly, genome quality was checked with GTDB-Tk (ver. 2.07) (1) and CheckM2 (ver. 1.0) (2) (**Table S1**). To assess the taxonomic position of KO_240205 and KO_240413, we analyzed their assemblies together with 227 reference genomes downloaded from the NCBI database, including *Mycobacterium* sp. strain MFM001 (**Table S2**). We annotated each genome with dfast-core under default settings (ver. 1.0.3) (3). The resulting gene annotations (GFF3 format) were analyzed with Roary software using the “-cd 100” option (ver. 3.11.2) (4) to compute core-gene alignments for all isolates. From the core-gene alignment, we estimated a maximum-likelihood tree with IQ-TREE 2 and the “-m MFP -bb 1000 -alrt 1000” options (ver. 2.2.0.3) (5). Average nucleotide identity (ANI) values among all *Mycobacterium* isolates were calculated with fastANI under default settings (ver. 1.31) (6). Digital DNA–DNA hybridization (dDDH) values were calculated with the Genome-to-Genome Distance Calculator (GGDC 3.0; DSMZ), based on the Genome BLAST Distance Phylogeny (GBDP) approach. We interpreted the values obtained with formula 2 (d4), which is recommended for incomplete draft genomes. We used a dDDH value of 70% as the species-delineation threshold (7).

| **Table S1**. Genome assembly statistics for KO_240205 and KO_240413 sequenced in this study | | | | | | |
| --- | --- | --- | --- | --- | --- | --- |
| Name | Completeness | Contamination | Contig_N50 | Genome_Size | GC_Content | classification by GTDB-Tk |
| KO_240205 | 99.97 | 0.09 | 481586 | 5016182 | 0.71 | d__Bacteria;p__Actinobacteriota;c__Actinomycetia;o__Mycobacteriales;  f__Mycobacteriaceae;g__Mycobacterium;s__Mycobacterium sp003402475 |
| KO_240413 | 99.97 | 0.09 | 758827 | 5014490 | 0.71 | d__Bacteria;p__Actinobacteriota;c__Actinomycetia;o__Mycobacteriales;  f__Mycobacteriaceae;g__Mycobacterium;s__Mycobacterium sp003402475 |

| **Table S2**. Genome assembly statistics for 227 *Mycobacterium* reference genomes available in the NCBI database | | | | | | | |
| --- | --- | --- | --- | --- | --- | --- | --- |
| Name | Completeness | Contamination | Contig_N50 | Genome_  Size | | GC_  Content | classification by GTDB-Tk |
| GCF_000015305.1 | 100 | 0.25 | 6491865 | 6491865 | 0.68 | | d__Bacteria;p__Actinobacteriota;c__Actinomycetia;o__Mycobacteriales;f__Mycobacteriaceae;g__Mycobacterium;s__Mycobacterium austroafricanum |
| GCF_000069185.1 | 100 | 0.14 | 5067172 | 5090491 | 0.68 | | d__Bacteria;p__Actinobacteriota;c__Actinomycetia;o__Mycobacteriales;f__Mycobacteriaceae;g__Mycobacterium;s__Mycobacterium abscessus |
| GCF_000157895.3 | 99.99 | 0.03 | 6432277 | 6577228 | 0.66 | | d__Bacteria;p__Actinobacteriota;c__Actinomycetia;o__Mycobacteriales;f__Mycobacteriaceae;g__Mycobacterium;s__Mycobacterium kansasii |
| GCF_000164135.1 | 99.99 | 0.14 | 1277640 | 6564171 | 0.7 | | d__Bacteria;p__Actinobacteriota;c__Actinomycetia;o__Mycobacteriales;f__Mycobacteriaceae;g__Mycobacterium;s__Mycobacterium parascrofulaceum |
| GCF_000184435.1 | 100 | 0.17 | 5547747 | 5783292 | 0.64 | | d__Bacteria;p__Actinobacteriota;c__Actinomycetia;o__Mycobacteriales;f__Mycobacteriaceae;g__Mycobacterium;s__Mycobacterium gilvum |
| GCF_000195955.2 | 100 | 0.75 | 4411532 | 4411532 | 0.66 | | d__Bacteria;p__Actinobacteriota;c__Actinomycetia;o__Mycobacteriales;f__Mycobacteriaceae;g__Mycobacterium;s__Mycobacterium tuberculosis |
| GCF_000214155.1 | 100 | 0.03 | 4643668 | 4643668 | 0.68 | | d__Bacteria;p__Actinobacteriota;c__Actinomycetia;o__Mycobacteriales;f__Mycobacteriaceae;g__Mycobacterium;s__Mycobacterium algericum |
| GCF_000222105.3 | 100 | 0.13 | 745349 | 5579559 | 0.69 | | d__Bacteria;p__Actinobacteriota;c__Actinomycetia;o__Mycobacteriales;f__Mycobacteriaceae;g__Mycobacterium;s__Mycobacterium colombiense |
| GCF_000253375.1 | 100 | 0.22 | 4482059 | 4482059 | 0.66 | | d__Bacteria;p__Actinobacteriota;c__Actinomycetia;o__Mycobacteriales;f__Mycobacteriaceae;g__Mycobacterium;s__Mycobacterium tuberculosis |
| GCF_000276825.1 | 100 | 0.26 | 5501090 | 5501090 | 0.68 | | d__Bacteria;p__Actinobacteriota;c__Actinomycetia;o__Mycobacteriales;f__Mycobacteriaceae;g__Mycobacterium;s__Mycobacterium intracellulare |
| GCF_000317305.3 | 100 | 0.01 | 5421267 | 5421267 | 0.67 | | d__Bacteria;p__Actinobacteriota;c__Actinomycetia;o__Mycobacteriales;f__Mycobacteriaceae;g__Mycobacterium;s__Mycobacterium neoaurum_A |
| GCF_000340435.2 | 99.94 | 0.11 | 4235765 | 4235765 | 0.64 | | d__Bacteria;p__Actinobacteriota;c__Actinomycetia;o__Mycobacteriales;f__Mycobacteriaceae;g__Mycobacterium;s__Mycobacterium haemophilum |
| GCF_000526915.1 | 99.9 | 0.01 | 465365 | 4936071 | 0.68 | | d__Bacteria;p__Actinobacteriota;c__Actinomycetia;o__Mycobacteriales;f__Mycobacteriaceae;g__Mycobacterium;s__Mycobacterium genavense |
| GCF_000559085.1 | 100 | 0.34 | 5494915 | 6297623 | 0.63 | | d__Bacteria;p__Actinobacteriota;c__Actinomycetia;o__Mycobacteriales;f__Mycobacteriaceae;g__Mycobacterium;s__Mycobacterium aromaticivorans |
| GCF_000613185.1 | 100 | 0.02 | 2465234 | 6462090 | 0.45 | | d__Bacteria;p__Actinobacteriota;c__Actinomycetia;o__Mycobacteriales;f__Mycobacteriaceae;g__Mycobacterium;s__Mycobacterium cosmeticum |
| GCF_000723425.2 | 100 | 0.45 | 6336293 | 6450522 | 0.64 | | d__Bacteria;p__Actinobacteriota;c__Actinomycetia;o__Mycobacteriales;f__Mycobacteriaceae;g__Mycobacterium;s__Mycobacterium marinum |
| GCF_000805375.1 | 100 | 0 | 891313 | 6065527 | 0.67 | | d__Bacteria;p__Actinobacteriota;c__Actinomycetia;o__Mycobacteriales;f__Mycobacteriaceae;g__Mycobacterium;s__Mycobacterium setense |
| GCF_000975265.2 | 83.13 | 0.18 | 3271694 | 3271694 | 0.58 | | d__Bacteria;p__Actinobacteriota;c__Actinomycetia;o__Mycobacteriales;f__Mycobacteriaceae;g__Mycobacterium;s__Mycobacterium lepromatosis |
| GCF_001050015.1 | 99.98 | 0.1 | 69270 | 5470965 | 0.68 | | d__Bacteria;p__Actinobacteriota;c__Actinomycetia;o__Mycobacteriales;f__Mycobacteriaceae;g__Mycobacterium;s__Mycobacterium malmesburyense |
| GCF_001050035.1 | 100 | 0 | 233711 | 5378970 | 0.67 | | d__Bacteria;p__Actinobacteriota;c__Actinomycetia;o__Mycobacteriales;f__Mycobacteriaceae;g__Mycobacterium;s__Mycobacterium komanii |
| GCF_001245615.1 | 100 | 0.07 | 3252791 | 6287317 | 0.68 | | d__Bacteria;p__Actinobacteriota;c__Actinomycetia;o__Mycobacteriales;f__Mycobacteriaceae;g__Mycobacterium;s__Mycobacterium neworleansense |
| GCF_001456355.1 | 99.98 | 0.05 | 4649175 | 4649175 | 0.65 | | d__Bacteria;p__Actinobacteriota;c__Actinomycetia;o__Mycobacteriales;f__Mycobacteriaceae;g__Mycobacterium;s__Mycobacterium saopaulense |
| GCF_001499835.1 | 100 | 1.06 | 182998 | 5702270 | 0.68 | | d__Bacteria;p__Actinobacteriota;c__Actinomycetia;o__Mycobacteriales;f__Mycobacteriaceae;g__Mycobacterium;s__Mycobacterium novocastrense |
| GCF_001499925.1 | 100 | 0 | 198352 | 5130549 | 0.66 | | d__Bacteria;p__Actinobacteriota;c__Actinomycetia;o__Mycobacteriales;f__Mycobacteriaceae;g__Mycobacterium;s__Mycobacterium lehmannii |
| GCF_001552315.1 | 100 | 1.41 | 265869 | 7330216 | 0.63 | | d__Bacteria;p__Actinobacteriota;c__Actinomycetia;o__Mycobacteriales;f__Mycobacteriaceae;g__Mycobacterium;s__Mycobacterium chlorophenolicum |
| GCF_001570425.1 | 100 | 0.53 | 262033 | 7387494 | 0.66 | | d__Bacteria;p__Actinobacteriota;c__Actinomycetia;o__Mycobacteriales;f__Mycobacteriaceae;g__Mycobacterium;s__Mycobacterium brisbanense |
| GCF_001605725.1 | 100 | 0.13 | 5573781 | 5573781 | 0.64 | | d__Bacteria;p__Actinobacteriota;c__Actinomycetia;o__Mycobacteriales;f__Mycobacteriaceae;g__Mycobacterium;s__Mycobacterium immunogenum |
| GCF_001632805.1 | 100 | 0.09 | 5029817 | 5029817 | 0.64 | | d__Bacteria;p__Actinobacteriota;c__Actinomycetia;o__Mycobacteriales;f__Mycobacteriaceae;g__Mycobacterium;s__Mycobacterium chelonae |
| GCF_001644575.1 | 100 | 0.57 | 5801417 | 6053985 | 0.66 | | d__Bacteria;p__Actinobacteriota;c__Actinomycetia;o__Mycobacteriales;f__Mycobacteriaceae;g__Mycobacterium;s__Mycobacterium sp001644575 |
| GCF_001655245.1 | 100 | 0.03 | 6235754 | 6235754 | 0.69 | | d__Bacteria;p__Actinobacteriota;c__Actinomycetia;o__Mycobacteriales;f__Mycobacteriaceae;g__Mycobacterium;s__Mycobacterium vaccae |
| GCF_001667535.1 | 100 | 0.01 | 198773 | 5223216 | 0.68 | | d__Bacteria;p__Actinobacteriota;c__Actinomycetia;o__Mycobacteriales;f__Mycobacteriaceae;g__Mycobacterium;s__Mycobacterium elephantis |
| GCF_001853795.1 | 100 | 0.69 | 1263333 | 5023635 | 0.61 | | d__Bacteria;p__Actinobacteriota;c__Actinomycetia;o__Mycobacteriales;f__Mycobacteriaceae;g__Mycobacterium;s__Mycobacterium franklinii |
| GCF_001854525.1 | 100 | 1.93 | 24990 | 5622166 | 0.76 | | d__Bacteria;p__Actinobacteriota;c__Actinomycetia;o__Mycobacteriales;f__Mycobacteriaceae;g__Mycobacterium;s__Mycobacterium eburneum |
| GCF_001942625.1 | 100 | 0.14 | 191633 | 6344489 | 0.65 | | d__Bacteria;p__Actinobacteriota;c__Actinomycetia;o__Mycobacteriales;f__Mycobacteriaceae;g__Mycobacterium;s__Mycobacterium syngnathidarum |
| GCF_002086115.1 | 100 | 0.01 | 203576 | 5952041 | 0.68 | | d__Bacteria;p__Actinobacteriota;c__Actinomycetia;o__Mycobacteriales;f__Mycobacteriaceae;g__Mycobacterium;s__Mycobacterium bacteremicum |
| GCF_002086125.1 | 100 | 0.82 | 52657 | 5980206 | 0.65 | | d__Bacteria;p__Actinobacteriota;c__Actinomycetia;o__Mycobacteriales;f__Mycobacteriaceae;g__Mycobacterium;s__Mycobacterium arosiense |
| GCF_002086165.1 | 100 | 0.07 | 95182 | 5897311 | 0.69 | | d__Bacteria;p__Actinobacteriota;c__Actinomycetia;o__Mycobacteriales;f__Mycobacteriaceae;g__Mycobacterium;s__Mycobacterium avium |
| GCF_002086285.1 | 100 | 0.37 | 110718 | 4815853 | 0.68 | | d__Bacteria;p__Actinobacteriota;c__Actinomycetia;o__Mycobacteriales;f__Mycobacteriaceae;g__Mycobacterium;s__Mycobacterium kumamotonense |
| GCF_002086485.1 | 100 | 1.18 | 135946 | 7927592 | 0.67 | | d__Bacteria;p__Actinobacteriota;c__Actinomycetia;o__Mycobacteriales;f__Mycobacteriaceae;g__Mycobacterium;s__Mycobacterium aquaticum |
| GCF_002086515.1 | 100 | 0 | 73139 | 4442590 | 0.67 | | d__Bacteria;p__Actinobacteriota;c__Actinomycetia;o__Mycobacteriales;f__Mycobacteriaceae;g__Mycobacterium;s__Mycobacterium arupense |
| GCF_002086545.1 | 100 | 0.4 | 118100 | 5969538 | 0.67 | | d__Bacteria;p__Actinobacteriota;c__Actinomycetia;o__Mycobacteriales;f__Mycobacteriaceae;g__Mycobacterium;s__Mycobacterium asiaticum |
| GCF_002086635.1 | 100 | 0.11 | 76856 | 5692845 | 0.7 | | d__Bacteria;p__Actinobacteriota;c__Actinomycetia;o__Mycobacteriales;f__Mycobacteriaceae;g__Mycobacterium;s__Mycobacterium alsense |
| GCF_002086695.1 | 100 | 0 | 325316 | 6009003 | 0.67 | | d__Bacteria;p__Actinobacteriota;c__Actinomycetia;o__Mycobacteriales;f__Mycobacteriaceae;g__Mycobacterium;s__Mycobacterium rhodesiae |
| GCF_002086735.1 | 100 | 0.01 | 99219 | 6170273 | 0.7 | | d__Bacteria;p__Actinobacteriota;c__Actinomycetia;o__Mycobacteriales;f__Mycobacteriaceae;g__Mycobacterium;s__Mycobacterium scrofulaceum |
| GCF_002086795.1 | 100 | 0.05 | 357339 | 6162880 | 0.65 | | d__Bacteria;p__Actinobacteriota;c__Actinomycetia;o__Mycobacteriales;f__Mycobacteriaceae;g__Mycobacterium;s__Mycobacterium tusciae |
| GCF_002086915.1 | 99.99 | 1.15 | 6254980 | 6347217 | 0.66 | | d__Bacteria;p__Actinobacteriota;c__Actinomycetia;o__Mycobacteriales;f__Mycobacteriaceae;g__Mycobacterium;s__Mycobacterium persicum |
| GCF_002101555.1 | 100 | 0.5 | 121943 | 6755573 | 0.67 | | d__Bacteria;p__Actinobacteriota;c__Actinomycetia;o__Mycobacteriales;f__Mycobacteriaceae;g__Mycobacterium;s__Mycobacterium canariasense |
| GCF_002101585.1 | 100 | 0.05 | 132590 | 4521435 | 0.64 | | d__Bacteria;p__Actinobacteriota;c__Actinomycetia;o__Mycobacteriales;f__Mycobacteriaceae;g__Mycobacterium;s__Mycobacterium engbaekii |
| GCF_002101595.1 | 99.98 | 0.04 | 162031 | 4720680 | 0.69 | | d__Bacteria;p__Actinobacteriota;c__Actinomycetia;o__Mycobacteriales;f__Mycobacteriaceae;g__Mycobacterium;s__Mycobacterium celatum |
| GCF_002101675.1 | 100 | 0.59 | 62676 | 7601632 | 0.67 | | d__Bacteria;p__Actinobacteriota;c__Actinomycetia;o__Mycobacteriales;f__Mycobacteriaceae;g__Mycobacterium;s__Mycobacterium gordonae |
| GCF_002101705.1 | 100 | 0.02 | 240675 | 6335651 | 0.67 | | d__Bacteria;p__Actinobacteriota;c__Actinomycetia;o__Mycobacteriales;f__Mycobacteriaceae;g__Mycobacterium;s__Mycobacterium iranicum |
| GCF_002101735.1 | 100 | 0.08 | 71978 | 5606671 | 0.69 | | d__Bacteria;p__Actinobacteriota;c__Actinomycetia;o__Mycobacteriales;f__Mycobacteriaceae;g__Mycobacterium;s__Mycobacterium kyorinense |
| GCF_002101775.1 | 100 | 0.03 | 138127 | 4465329 | 0.68 | | d__Bacteria;p__Actinobacteriota;c__Actinomycetia;o__Mycobacteriales;f__Mycobacteriaceae;g__Mycobacterium;s__Mycobacterium nonchromogenicum |
| GCF_002101785.1 | 100 | 6.32 | 85200 | 6037522 | 0.71 | | d__Bacteria;p__Actinobacteriota;c__Actinomycetia;o__Mycobacteriales;f__Mycobacteriaceae;g__Mycobacterium;s__Mycobacterium palustre |
| GCF_002101815.1 | 100 | 0 | 271458 | 5619528 | 0.67 | | d__Bacteria;p__Actinobacteriota;c__Actinomycetia;o__Mycobacteriales;f__Mycobacteriaceae;g__Mycobacterium;s__Mycobacterium paraense |
| GCF_002101965.1 | 100 | 0 | 481307 | 7516859 | 0.67 | | d__Bacteria;p__Actinobacteriota;c__Actinomycetia;o__Mycobacteriales;f__Mycobacteriaceae;g__Mycobacterium;s__Mycobacterium wolinskyi |
| GCF_002102025.1 | 100 | 0.1 | 220473 | 5420516 | 0.79 | | d__Bacteria;p__Actinobacteriota;c__Actinomycetia;o__Mycobacteriales;f__Mycobacteriaceae;g__Mycobacterium;s__Mycobacterium bohemicum |
| GCF_002102065.1 | 100 | 0 | 320022 | 6468817 | 0.62 | | d__Bacteria;p__Actinobacteriota;c__Actinomycetia;o__Mycobacteriales;f__Mycobacteriaceae;g__Mycobacterium;s__Mycobacterium farcinogenes |
| GCF_002102175.1 | 99.99 | 0 | 85262 | 5816659 | 0.66 | | d__Bacteria;p__Actinobacteriota;c__Actinomycetia;o__Mycobacteriales;f__Mycobacteriaceae;g__Mycobacterium;s__Mycobacterium gastri |
| GCF_002102185.1 | 100 | 0.03 | 284076 | 4731047 | 0.62 | | d__Bacteria;p__Actinobacteriota;c__Actinomycetia;o__Mycobacteriales;f__Mycobacteriaceae;g__Mycobacterium;s__Mycobacterium fragae |
| GCF_002102255.1 | 99.99 | 0.2 | 86703 | 6727177 | 0.65 | | d__Bacteria;p__Actinobacteriota;c__Actinomycetia;o__Mycobacteriales;f__Mycobacteriaceae;g__Mycobacterium;s__Mycobacterium nebraskense |
| GCF_002102265.1 | 100 | 0 | 159248 | 4812247 | 0.68 | | d__Bacteria;p__Actinobacteriota;c__Actinomycetia;o__Mycobacteriales;f__Mycobacteriaceae;g__Mycobacterium;s__Mycobacterium longobardum |
| GCF_002102345.1 | 100 | 0.08 | 201665 | 7050152 | 0.64 | | d__Bacteria;p__Actinobacteriota;c__Actinomycetia;o__Mycobacteriales;f__Mycobacteriaceae;g__Mycobacterium;s__Mycobacterium peregrinum |
| GCF_002102355.1 | 100 | 0.27 | 125930 | 5562166 | 0.69 | | d__Bacteria;p__Actinobacteriota;c__Actinomycetia;o__Mycobacteriales;f__Mycobacteriaceae;g__Mycobacterium;s__Mycobacterium sherrisii |
| GCF_002102395.1 | 100 | 0.21 | 127871 | 3591083 | 0.69 | | d__Bacteria;p__Actinobacteriota;c__Actinomycetia;o__Mycobacteriales;f__Mycobacteriaceae;g__Mycobacterium;s__Mycobacterium triviale |
| GCF_002102415.1 | 100 | 0.23 | 258006 | 6366285 | 0.68 | | d__Bacteria;p__Actinobacteriota;c__Actinomycetia;o__Mycobacteriales;f__Mycobacteriaceae;g__Mycobacterium;s__Mycobacterium triplex |
| GCF_002104675.1 | 100 | 0.2 | 114870 | 5225940 | 0.65 | | d__Bacteria;p__Actinobacteriota;c__Actinomycetia;o__Mycobacteriales;f__Mycobacteriaceae;g__Mycobacterium;s__Mycobacterium decipiens |
| GCF_002104765.1 | 100 | 0.49 | 154398 | 6266718 | 0.67 | | d__Bacteria;p__Actinobacteriota;c__Actinomycetia;o__Mycobacteriales;f__Mycobacteriaceae;g__Mycobacterium;s__Mycobacterium vulneris |
| GCF_002116635.1 | 99.95 | 0.33 | 95710 | 6672659 | 0.65 | | d__Bacteria;p__Actinobacteriota;c__Actinomycetia;o__Mycobacteriales;f__Mycobacteriaceae;g__Mycobacterium;s__Mycobacterium szulgai |
| GCF_002157835.1 | 100 | 1.06 | 7595921 | 8080416 | 0.66 | | d__Bacteria;p__Actinobacteriota;c__Actinomycetia;o__Mycobacteriales;f__Mycobacteriaceae;g__Mycobacterium;s__Mycobacterium dioxanotrophicus |
| GCF_002245615.1 | 100 | 0 | 200051 | 5376391 | 0.65 | | d__Bacteria;p__Actinobacteriota;c__Actinomycetia;o__Mycobacteriales;f__Mycobacteriaceae;g__Mycobacterium;s__Mycobacterium flavescens |
| GCF_002250655.1 | 100 | 0.15 | 167586 | 6066978 | 0.66 | | d__Bacteria;p__Actinobacteriota;c__Actinomycetia;o__Mycobacteriales;f__Mycobacteriaceae;g__Mycobacterium;s__Mycobacterium sphagni |
| GCF_002285715.1 | 100 | 0.01 | 5266037 | 5285642 | 0.64 | | d__Bacteria;p__Actinobacteriota;c__Actinomycetia;o__Mycobacteriales;f__Mycobacteriaceae;g__Mycobacterium;s__Mycobacterium marseillense |
| GCF_002355775.1 | 99.99 | 1.8 | 5899681 | 6066298 | 0.63 | | d__Bacteria;p__Actinobacteriota;c__Actinomycetia;o__Mycobacteriales;f__Mycobacteriaceae;g__Mycobacterium;s__Mycobacterium marinum |
| GCF_002356315.1 | 100 | 0 | 5232660 | 5232660 | 0.67 | | d__Bacteria;p__Actinobacteriota;c__Actinomycetia;o__Mycobacteriales;f__Mycobacteriaceae;g__Mycobacterium;s__Mycobacterium shigaense |
| GCF_002591975.1 | 100 | 0.1 | 116368 | 6209311 | 0.67 | | d__Bacteria;p__Actinobacteriota;c__Actinomycetia;o__Mycobacteriales;f__Mycobacteriaceae;g__Mycobacterium;s__Mycobacterium neglectum |
| GCF_002592005.1 | 100 | 2.2 | 61684 | 6230283 | 0.68 | | d__Bacteria;p__Actinobacteriota;c__Actinomycetia;o__Mycobacteriales;f__Mycobacteriaceae;g__Mycobacterium;s__Mycobacterium palauense |
| GCF_002838065.1 | 96.32 | 0.06 | 117476 | 5575795 | 0.69 | | d__Bacteria;p__Actinobacteriota;c__Actinomycetia;o__Mycobacteriales;f__Mycobacteriaceae;g__Mycobacterium;s__Mycobacterium sp002838065 |
| GCF_003112775.1 | 100 | 0.02 | 416792 | 5744567 | 0.56 | | d__Bacteria;p__Actinobacteriota;c__Actinomycetia;o__Mycobacteriales;f__Mycobacteriaceae;g__Mycobacterium;s__Mycobacterium montefiorense |
| GCF_003253775.1 | 91.43 | 0.14 | 3187112 | 3187112 | 0.58 | | d__Bacteria;p__Actinobacteriota;c__Actinomycetia;o__Mycobacteriales;f__Mycobacteriaceae;g__Mycobacterium;s__Mycobacterium leprae |
| GCF_003254575.1 | 99.99 | 0.05 | 102117 | 5759757 | 0.67 | | d__Bacteria;p__Actinobacteriota;c__Actinomycetia;o__Mycobacteriales;f__Mycobacteriaceae;g__Mycobacterium;s__Mycobacterium obuense |
| GCF_003408705.1 | 99.69 | 0.14 | 121366 | 3122721 | 0.59 | | d__Bacteria;p__Actinobacteriota;c__Actinomycetia;o__Mycobacteriales;f__Mycobacteriaceae;g__Mycobacterium;s__Mycobacterium uberis |
| GCF_003584745.1 | 100 | 0.75 | 6061597 | 6061597 | 0.66 | | d__Bacteria;p__Actinobacteriota;c__Actinomycetia;o__Mycobacteriales;f__Mycobacteriaceae;g__Mycobacterium;s__Mycobacterium marinum |
| GCF_003614435.1 | 100 | 0.34 | 6730319 | 7224251 | 0.67 | | d__Bacteria;p__Actinobacteriota;c__Actinomycetia;o__Mycobacteriales;f__Mycobacteriaceae;g__Mycobacterium;s__Mycobacterium paragordonae |
| GCF_003719305.1 | 100 | 0.36 | 175888 | 6956345 | 0.67 | | d__Bacteria;p__Actinobacteriota;c__Actinomycetia;o__Mycobacteriales;f__Mycobacteriaceae;g__Mycobacterium;s__Mycobacterium sp003719305 |
| GCF_003855255.1 | 100 | 0.11 | 6905961 | 6905961 | 0.67 | | d__Bacteria;p__Actinobacteriota;c__Actinomycetia;o__Mycobacteriales;f__Mycobacteriaceae;g__Mycobacterium;s__Mycobacterium septicum |
| GCF_004359005.1 | 100 | 0.04 | 624616 | 5599206 | 0.67 | | d__Bacteria;p__Actinobacteriota;c__Actinomycetia;o__Mycobacteriales;f__Mycobacteriaceae;g__Mycobacterium;s__Mycobacterium obuense |
| GCF_004924335.1 | 100 | 0.07 | 4776697 | 4776697 | 0.64 | | d__Bacteria;p__Actinobacteriota;c__Actinomycetia;o__Mycobacteriales;f__Mycobacteriaceae;g__Mycobacterium;s__Mycobacterium salmoniphilum |
| GCF_005670685.2 | 100 | 0.25 | 6099273 | 6099273 | 0.67 | | d__Bacteria;p__Actinobacteriota;c__Actinomycetia;o__Mycobacteriales;f__Mycobacteriaceae;g__Mycobacterium;s__Mycobacterium mucogenicum_B |
| GCF_007714185.1 | 99.95 | 0.32 | 42257 | 5772032 | 0.64 | | d__Bacteria;p__Actinobacteriota;c__Actinomycetia;o__Mycobacteriales;f__Mycobacteriaceae;g__Mycobacterium;s__Mycobacterium helveticum |
| GCF_008329645.1 | 100 | 0 | 5637923 | 5681602 | 0.66 | | d__Bacteria;p__Actinobacteriota;c__Actinomycetia;o__Mycobacteriales;f__Mycobacteriaceae;g__Mycobacterium;s__Mycobacterium grossiae |
| GCF_010722915.1 | 99.99 | 2.53 | 6077648 | 7297392 | 0.66 | | d__Bacteria;p__Actinobacteriota;c__Actinomycetia;o__Mycobacteriales;f__Mycobacteriaceae;g__Mycobacterium;s__Mycobacterium agri |
| GCF_010722995.1 | 100 | 0.63 | 6743693 | 6838280 | 0.68 | | d__Bacteria;p__Actinobacteriota;c__Actinomycetia;o__Mycobacteriales;f__Mycobacteriaceae;g__Mycobacterium;s__Mycobacterium tokaiense |
| GCF_010723225.1 | 99.95 | 1.18 | 2700447 | 4611963 | 0.68 | | d__Bacteria;p__Actinobacteriota;c__Actinomycetia;o__Mycobacteriales;f__Mycobacteriaceae;g__Mycobacterium;s__Mycobacterium senuense |
| GCF_010723305.1 | 99.83 | 0.29 | 2188254 | 4335050 | 0.66 | | d__Bacteria;p__Actinobacteriota;c__Actinomycetia;o__Mycobacteriales;f__Mycobacteriaceae;g__Mycobacterium;s__Mycobacterium botniense |
| GCF_010723515.1 | 100 | 0 | 4721446 | 4756495 | 0.61 | | d__Bacteria;p__Actinobacteriota;c__Actinomycetia;o__Mycobacteriales;f__Mycobacteriaceae;g__Mycobacterium;s__Mycobacterium algericum |
| GCF_010723575.1 | 100 | 0.07 | 5817465 | 6912804 | 0.66 | | d__Bacteria;p__Actinobacteriota;c__Actinomycetia;o__Mycobacteriales;f__Mycobacteriaceae;g__Mycobacterium;s__Mycobacterium bourgelatii |
| GCF_010723735.1 | 99.99 | 0.72 | 5948177 | 6160733 | 0.69 | | d__Bacteria;p__Actinobacteriota;c__Actinomycetia;o__Mycobacteriales;f__Mycobacteriaceae;g__Mycobacterium;s__Mycobacterium hippocampi_B |
| GCF_010725485.1 | 100 | 0.03 | 6044196 | 6044196 | 0.68 | | d__Bacteria;p__Actinobacteriota;c__Actinomycetia;o__Mycobacteriales;f__Mycobacteriaceae;g__Mycobacterium;s__Mycobacterium sp002946335 |
| GCF_010725725.1 | 99.99 | 0.11 | 5484749 | 5484749 | 0.68 | | d__Bacteria;p__Actinobacteriota;c__Actinomycetia;o__Mycobacteriales;f__Mycobacteriaceae;g__Mycobacterium;s__Mycobacterium pulveris |
| GCF_010725885.1 | 100 | 0.19 | 6328149 | 6328149 | 0.68 | | d__Bacteria;p__Actinobacteriota;c__Actinomycetia;o__Mycobacteriales;f__Mycobacteriaceae;g__Mycobacterium;s__Mycobacterium tokaiense |
| GCF_010726085.1 | 100 | 0.26 | 6225779 | 6225779 | 0.66 | | d__Bacteria;p__Actinobacteriota;c__Actinomycetia;o__Mycobacteriales;f__Mycobacteriaceae;g__Mycobacterium;s__Mycobacterium moriokaense |
| GCF_010726245.1 | 100 | 0.13 | 5925482 | 5925482 | 0.67 | | d__Bacteria;p__Actinobacteriota;c__Actinomycetia;o__Mycobacteriales;f__Mycobacteriaceae;g__Mycobacterium;s__Mycobacterium aichiense |
| GCF_010726505.1 | 100 | 1.05 | 4458926 | 4458926 | 0.69 | | d__Bacteria;p__Actinobacteriota;c__Actinomycetia;o__Mycobacteriales;f__Mycobacteriaceae;g__Mycobacterium;s__Mycobacterium algericum |
| GCF_010726645.1 | 100 | 0.01 | 5657388 | 5657388 | 0.68 | | d__Bacteria;p__Actinobacteriota;c__Actinomycetia;o__Mycobacteriales;f__Mycobacteriaceae;g__Mycobacterium;s__Mycobacterium duvalii |
| GCF_010726765.1 | 100 | 0.34 | 6090007 | 6301681 | 0.65 | | d__Bacteria;p__Actinobacteriota;c__Actinomycetia;o__Mycobacteriales;f__Mycobacteriaceae;g__Mycobacterium;s__Mycobacterium gallinarum |
| GCF_010726955.1 | 100 | 0.33 | 4156821 | 4156821 | 0.7 | | d__Bacteria;p__Actinobacteriota;c__Actinomycetia;o__Mycobacteriales;f__Mycobacteriaceae;g__Mycobacterium;s__Mycobacterium fallax |
| GCF_010727125.1 | 100 | 0 | 4566186 | 4566186 | 0.68 | | d__Bacteria;p__Actinobacteriota;c__Actinomycetia;o__Mycobacteriales;f__Mycobacteriaceae;g__Mycobacterium;s__Mycobacterium terrae |
| GCF_010727325.1 | 100 | 1.35 | 5712683 | 6016846 | 0.64 | | d__Bacteria;p__Actinobacteriota;c__Actinomycetia;o__Mycobacteriales;f__Mycobacteriaceae;g__Mycobacterium;s__Mycobacterium alvei |
| GCF_010727475.1 | 100 | 0.23 | 8006721 | 8006721 | 0.67 | | d__Bacteria;p__Actinobacteriota;c__Actinomycetia;o__Mycobacteriales;f__Mycobacteriaceae;g__Mycobacterium;s__Mycobacterium mageritense |
| GCF_010727605.1 | 100 | 0.21 | 5788994 | 5788994 | 0.66 | | d__Bacteria;p__Actinobacteriota;c__Actinomycetia;o__Mycobacteriales;f__Mycobacteriaceae;g__Mycobacterium;s__Mycobacterium simiae |
| GCF_010727945.1 | 99.98 | 1.22 | 5318517 | 5318517 | 0.66 | | d__Bacteria;p__Actinobacteriota;c__Actinomycetia;o__Mycobacteriales;f__Mycobacteriaceae;g__Mycobacterium;s__Mycobacterium cookii |
| GCF_010728155.1 | 100 | 0.18 | 4029398 | 4029398 | 0.68 | | d__Bacteria;p__Actinobacteriota;c__Actinomycetia;o__Mycobacteriales;f__Mycobacteriaceae;g__Mycobacterium;s__Mycobacterium doricum |
| GCF_010728325.1 | 100 | 0.07 | 5712830 | 5929171 | 0.66 | | d__Bacteria;p__Actinobacteriota;c__Actinomycetia;o__Mycobacteriales;f__Mycobacteriaceae;g__Mycobacterium;s__Mycobacterium poriferae |
| GCF_010728525.1 | 100 | 0.27 | 5973149 | 5973149 | 0.66 | | d__Bacteria;p__Actinobacteriota;c__Actinomycetia;o__Mycobacteriales;f__Mycobacteriaceae;g__Mycobacterium;s__Mycobacterium marinum |
| GCF_010728725.1 | 100 | 0.6 | 5115366 | 5979623 | 0.65 | | d__Bacteria;p__Actinobacteriota;c__Actinomycetia;o__Mycobacteriales;f__Mycobacteriaceae;g__Mycobacterium;s__Mycobacterium branderi |
| GCF_010728925.1 | 99.99 | 0.24 | 5964999 | 5964999 | 0.66 | | d__Bacteria;p__Actinobacteriota;c__Actinomycetia;o__Mycobacteriales;f__Mycobacteriaceae;g__Mycobacterium;s__Mycobacterium gadium |
| GCF_010729105.1 | 100 | 1.28 | 6008916 | 6008916 | 0.68 | | d__Bacteria;p__Actinobacteriota;c__Actinomycetia;o__Mycobacteriales;f__Mycobacteriaceae;g__Mycobacterium;s__Mycobacterium saskatchewanense |
| GCF_010729305.1 | 100 | 0.01 | 5732362 | 5732362 | 0.69 | | d__Bacteria;p__Actinobacteriota;c__Actinomycetia;o__Mycobacteriales;f__Mycobacteriaceae;g__Mycobacterium;s__Mycobacterium psychrotolerans |
| GCF_010729485.1 | 100 | 0.03 | 4396654 | 4396654 | 0.69 | | d__Bacteria;p__Actinobacteriota;c__Actinomycetia;o__Mycobacteriales;f__Mycobacteriaceae;g__Mycobacterium;s__Mycobacterium hiberniae |
| GCF_010729665.1 | 99.92 | 1.04 | 5712088 | 5940375 | 0.66 | | d__Bacteria;p__Actinobacteriota;c__Actinomycetia;o__Mycobacteriales;f__Mycobacteriaceae;g__Mycobacterium;s__Mycobacterium madagascariense |
| GCF_010729895.1 | 100 | 0.03 | 5876557 | 5876557 | 0.67 | | d__Bacteria;p__Actinobacteriota;c__Actinomycetia;o__Mycobacteriales;f__Mycobacteriaceae;g__Mycobacterium;s__Mycobacterium confluentis |
| GCF_010730055.1 | 100 | 0.18 | 4504020 | 4504020 | 0.68 | | d__Bacteria;p__Actinobacteriota;c__Actinomycetia;o__Mycobacteriales;f__Mycobacteriaceae;g__Mycobacterium;s__Mycobacterium shinjukuense |
| GCF_010730195.1 | 100 | 0.03 | 6237139 | 6237139 | 0.67 | | d__Bacteria;p__Actinobacteriota;c__Actinomycetia;o__Mycobacteriales;f__Mycobacteriaceae;g__Mycobacterium;s__Mycobacterium conspicuum |
| GCF_010730355.1 | 100 | 0.12 | 6219859 | 6219859 | 0.66 | | d__Bacteria;p__Actinobacteriota;c__Actinomycetia;o__Mycobacteriales;f__Mycobacteriaceae;g__Mycobacterium;s__Mycobacterium florentinum |
| GCF_010730575.1 | 100 | 0.07 | 5952912 | 5952912 | 0.68 | | d__Bacteria;p__Actinobacteriota;c__Actinomycetia;o__Mycobacteriales;f__Mycobacteriaceae;g__Mycobacterium;s__Mycobacterium parmense |
| GCF_010730745.1 | 100 | 0.04 | 5050576 | 5050576 | 0.68 | | d__Bacteria;p__Actinobacteriota;c__Actinomycetia;o__Mycobacteriales;f__Mycobacteriaceae;g__Mycobacterium;s__Mycobacterium heidelbergense |
| GCF_010730955.1 | 100 | 0.81 | 6055481 | 6321928 | 0.65 | | d__Bacteria;p__Actinobacteriota;c__Actinomycetia;o__Mycobacteriales;f__Mycobacteriaceae;g__Mycobacterium;s__Mycobacterium aubagnense |
| GCF_010731115.1 | 100 | 0.78 | 5853197 | 5853197 | 0.67 | | d__Bacteria;p__Actinobacteriota;c__Actinomycetia;o__Mycobacteriales;f__Mycobacteriaceae;g__Mycobacterium;s__Mycobacterium phocaicum |
| GCF_010731295.1 | 100 | 0.27 | 6563937 | 6585921 | 0.64 | | d__Bacteria;p__Actinobacteriota;c__Actinomycetia;o__Mycobacteriales;f__Mycobacteriaceae;g__Mycobacterium;s__Mycobacterium boenickei |
| GCF_010731535.1 | 100 | 0 | 5092988 | 5092988 | 0.67 | | d__Bacteria;p__Actinobacteriota;c__Actinomycetia;o__Mycobacteriales;f__Mycobacteriaceae;g__Mycobacterium;s__Mycobacterium lacus |
| GCF_010731575.1 | 100 | 0.34 | 6042466 | 6042466 | 0.68 | | d__Bacteria;p__Actinobacteriota;c__Actinomycetia;o__Mycobacteriales;f__Mycobacteriaceae;g__Mycobacterium;s__Mycobacterium monacense |
| GCF_010731595.1 | 99.98 | 0.28 | 5531300 | 5531300 | 0.68 | | d__Bacteria;p__Actinobacteriota;c__Actinomycetia;o__Mycobacteriales;f__Mycobacteriaceae;g__Mycobacterium;s__Mycobacterium seoulense |
| GCF_010731615.1 | 100 | 0.11 | 4577045 | 4577045 | 0.69 | | d__Bacteria;p__Actinobacteriota;c__Actinomycetia;o__Mycobacteriales;f__Mycobacteriaceae;g__Mycobacterium;s__Mycobacterium insubricum |
| GCF_010731635.1 | 100 | 0.03 | 4779798 | 4779798 | 0.66 | | d__Bacteria;p__Actinobacteriota;c__Actinomycetia;o__Mycobacteriales;f__Mycobacteriaceae;g__Mycobacterium;s__Mycobacterium noviomagense |
| GCF_010731655.1 | 100 | 0.28 | 6085955 | 6085955 | 0.68 | | d__Bacteria;p__Actinobacteriota;c__Actinomycetia;o__Mycobacteriales;f__Mycobacteriaceae;g__Mycobacterium;s__Mycobacterium paraseoulense |
| GCF_010731695.1 | 100 | 1.4 | 5579510 | 5579510 | 0.69 | | d__Bacteria;p__Actinobacteriota;c__Actinomycetia;o__Mycobacteriales;f__Mycobacteriaceae;g__Mycobacterium;s__Mycobacterium litorale |
| GCF_010731715.1 | 100 | 0.18 | 6210822 | 6210822 | 0.66 | | d__Bacteria;p__Actinobacteriota;c__Actinomycetia;o__Mycobacteriales;f__Mycobacteriaceae;g__Mycobacterium;s__Mycobacterium stomatepiae |
| GCF_010731735.1 | 100 | 0.16 | 6250641 | 6250641 | 0.69 | | d__Bacteria;p__Actinobacteriota;c__Actinomycetia;o__Mycobacteriales;f__Mycobacteriaceae;g__Mycobacterium;s__Mycobacterium sediminis |
| GCF_010731755.1 | 100 | 0.18 | 4224047 | 4224047 | 0.67 | | d__Bacteria;p__Actinobacteriota;c__Actinomycetia;o__Mycobacteriales;f__Mycobacteriaceae;g__Mycobacterium;s__Mycobacterium minnesotense |
| GCF_010731775.1 | 100 | 0.24 | 6185541 | 6185541 | 0.67 | | d__Bacteria;p__Actinobacteriota;c__Actinomycetia;o__Mycobacteriales;f__Mycobacteriaceae;g__Mycobacterium;s__Mycobacterium mantenii |
| GCF_010731795.1 | 100 | 0 | 5024249 | 5024249 | 0.67 | | d__Bacteria;p__Actinobacteriota;c__Actinomycetia;o__Mycobacteriales;f__Mycobacteriaceae;g__Mycobacterium;s__Mycobacterium celeriflavum |
| GCF_010731815.2 | 100 | 1.74 | 6017160 | 6451210 | 0.66 | | d__Bacteria;p__Actinobacteriota;c__Actinomycetia;o__Mycobacteriales;f__Mycobacteriaceae;g__Mycobacterium;s__Mycobacterium arabiense |
| GCF_010731835.1 | 100 | 0.63 | 4155701 | 4155701 | 0.69 | | d__Bacteria;p__Actinobacteriota;c__Actinomycetia;o__Mycobacteriales;f__Mycobacteriaceae;g__Mycobacterium;s__Mycobacterium koreense |
| GCF_010731855.1 | 100 | 0.1 | 5696751 | 5696751 | 0.67 | | d__Bacteria;p__Actinobacteriota;c__Actinomycetia;o__Mycobacteriales;f__Mycobacteriaceae;g__Mycobacterium;s__Mycobacterium anyangense |
| GCF_010731875.1 | 100 | 0.56 | 4828255 | 4828255 | 0.66 | | d__Bacteria;p__Actinobacteriota;c__Actinomycetia;o__Mycobacteriales;f__Mycobacteriaceae;g__Mycobacterium;s__Mycobacterium sarraceniae |
| GCF_010731895.1 | 100 | 0.26 | 6400811 | 6400811 | 0.66 | | d__Bacteria;p__Actinobacteriota;c__Actinomycetia;o__Mycobacteriales;f__Mycobacteriaceae;g__Mycobacterium;s__Mycobacterium helvum |
| GCF_012223425.1 | 99.99 | 0.34 | 6086872 | 6713618 | 0.66 | | d__Bacteria;p__Actinobacteriota;c__Actinomycetia;o__Mycobacteriales;f__Mycobacteriaceae;g__Mycobacterium;s__Mycobacterium frederiksbergense |
| GCF_013404075.1 | 100 | 0.42 | 127043 | 7196491 | 0.63 | | d__Bacteria;p__Actinobacteriota;c__Actinomycetia;o__Mycobacteriales;f__Mycobacteriaceae;g__Mycobacterium;s__Mycobacterium sp013404075 |
| GCF_013466425.1 | 100 | 2.09 | 6266765 | 6266765 | 0.65 | | d__Bacteria;p__Actinobacteriota;c__Actinomycetia;o__Mycobacteriales;f__Mycobacteriaceae;g__Mycobacterium;s__Mycobacterium vicinigordonae |
| GCF_014263315.1 | 100 | 0.11 | 5345311 | 5463479 | 0.65 | | d__Bacteria;p__Actinobacteriota;c__Actinomycetia;o__Mycobacteriales;f__Mycobacteriaceae;g__Mycobacterium;s__Mycobacterium kubicae |
| GCF_014295435.1 | 100 | 0.64 | 6837082 | 7117477 | 0.66 | | d__Bacteria;p__Actinobacteriota;c__Actinomycetia;o__Mycobacteriales;f__Mycobacteriaceae;g__Mycobacterium;s__Mycobacterium fluoranthenivorans |
| GCF_015710575.1 | 99.94 | 0.76 | 5962395 | 5962395 | 0.66 | | d__Bacteria;p__Actinobacteriota;c__Actinomycetia;o__Mycobacteriales;f__Mycobacteriaceae;g__Mycobacterium;s__Mycobacterium sp015710575 |
| GCF_016313185.1 | 99.75 | 0.04 | 6181092 | 6181092 | 0.68 | | d__Bacteria;p__Actinobacteriota;c__Actinomycetia;o__Mycobacteriales;f__Mycobacteriaceae;g__Mycobacterium;s__Mycobacterium sp016313185 |
| GCF_016861545.1 | 100 | 0.06 | 4865109 | 4865109 | 0.66 | | d__Bacteria;p__Actinobacteriota;c__Actinomycetia;o__Mycobacteriales;f__Mycobacteriaceae;g__Mycobacterium;s__Mycobacterium heckeshornense |
| GCF_016919345.1 | 100 | 0.15 | 6515693 | 6515693 | 0.67 | | d__Bacteria;p__Actinobacteriota;c__Actinomycetia;o__Mycobacteriales;f__Mycobacteriaceae;g__Mycobacterium;s__Mycobacterium septicum |
| GCF_016919645.1 | 100 | 0.05 | 6287627 | 6287627 | 0.68 | | d__Bacteria;p__Actinobacteriota;c__Actinomycetia;o__Mycobacteriales;f__Mycobacteriaceae;g__Mycobacterium;s__Mycobacterium austroafricanum |
| GCF_017876775.1 | 100 | 1.65 | 4375269 | 6345289 | 0.64 | | d__Bacteria;p__Actinobacteriota;c__Actinomycetia;o__Mycobacteriales;f__Mycobacteriaceae;g__Mycobacterium;s__Mycobacterium lutetiense |
| GCF_018278905.1 | 99.99 | 0.2 | 5581157 | 5581157 | 0.66 | | d__Bacteria;p__Actinobacteriota;c__Actinomycetia;o__Mycobacteriales;f__Mycobacteriaceae;g__Mycobacterium;s__Mycobacterium spongiae |
| GCF_018455725.1 | 100 | 0.06 | 90773 | 4545982 | 0.71 | | d__Bacteria;p__Actinobacteriota;c__Actinomycetia;o__Mycobacteriales;f__Mycobacteriaceae;g__Mycobacterium;s__Mycobacterium sp018455725 |
| GCF_019456655.1 | 100 | 0.29 | 5998503 | 5998503 | 0.68 | | d__Bacteria;p__Actinobacteriota;c__Actinomycetia;o__Mycobacteriales;f__Mycobacteriaceae;g__Mycobacterium;s__Mycobacterium diernhoferi |
| GCF_019456675.1 | 100 | 0.09 | 6035548 | 6093551 | 0.66 | | d__Bacteria;p__Actinobacteriota;c__Actinomycetia;o__Mycobacteriales;f__Mycobacteriaceae;g__Mycobacterium;s__Mycobacterium sp001886515 |
| GCF_019645815.1 | 100 | 0 | 5061737 | 5061737 | 0.68 | | d__Bacteria;p__Actinobacteriota;c__Actinomycetia;o__Mycobacteriales;f__Mycobacteriaceae;g__Mycobacterium;s__Mycobacterium heraklionense_B |
| GCF_019645835.1 | 99.99 | 0.21 | 5623714 | 5623714 | 0.67 | | d__Bacteria;p__Actinobacteriota;c__Actinomycetia;o__Mycobacteriales;f__Mycobacteriaceae;g__Mycobacterium;s__Mycobacterium holsaticum |
| GCF_019645855.1 | 100 | 0.4 | 5319859 | 5319859 | 0.67 | | d__Bacteria;p__Actinobacteriota;c__Actinomycetia;o__Mycobacteriales;f__Mycobacteriaceae;g__Mycobacterium;s__Mycobacterium malmoense_B |
| GCF_019645875.1 | 100 | 0 | 6086722 | 6086722 | 0.67 | | d__Bacteria;p__Actinobacteriota;c__Actinomycetia;o__Mycobacteriales;f__Mycobacteriaceae;g__Mycobacterium;s__Mycobacterium farcinogenes |
| GCF_019668465.1 | 100 | 0.18 | 5795031 | 5831451 | 0.66 | | d__Bacteria;p__Actinobacteriota;c__Actinomycetia;o__Mycobacteriales;f__Mycobacteriaceae;g__Mycobacterium;s__ |
| GCF_019710635.1 | 100 | 0 | 129365 | 5553058 | 0.67 | | d__Bacteria;p__Actinobacteriota;c__Actinomycetia;o__Mycobacteriales;f__Mycobacteriaceae;g__Mycobacterium;s__ |
| GCF_019739075.1 | 100 | 0.01 | 6344228 | 6607534 | 0.64 | | d__Bacteria;p__Actinobacteriota;c__Actinomycetia;o__Mycobacteriales;f__Mycobacteriaceae;g__Mycobacterium;s__Mycobacterium farcinogenes |
| GCF_020736005.1 | 100 | 0.35 | 5621211 | 5645778 | 0.65 | | d__Bacteria;p__Actinobacteriota;c__Actinomycetia;o__Mycobacteriales;f__Mycobacteriaceae;g__Mycobacterium;s__Mycobacterium intracellulare |
| GCF_021183725.1 | 100 | 0.07 | 6127734 | 6127734 | 0.66 | | d__Bacteria;p__Actinobacteriota;c__Actinomycetia;o__Mycobacteriales;f__Mycobacteriaceae;g__Mycobacterium;s__Mycobacterium ostraviense |
| GCF_021916785.1 | 100 | 0.08 | 596067 | 6138096 | 0.68 | | d__Bacteria;p__Actinobacteriota;c__Actinomycetia;o__Mycobacteriales;f__Mycobacteriaceae;g__Mycobacterium;s__ |
| GCF_022175585.2 | 100 | 0 | 5173839 | 5173839 | 0.69 | | d__Bacteria;p__Actinobacteriota;c__Actinomycetia;o__Mycobacteriales;f__Mycobacteriaceae;g__Mycobacterium;s__Mycobacterium avium |
| GCF_022179545.1 | 100 | 0.17 | 6406072 | 6406072 | 0.66 | | d__Bacteria;p__Actinobacteriota;c__Actinomycetia;o__Mycobacteriales;f__Mycobacteriaceae;g__Mycobacterium;s__Mycobacterium fortuitum |
| GCF_022370415.1 | 100 | 0.52 | 6895172 | 6895172 | 0.67 | | d__Bacteria;p__Actinobacteriota;c__Actinomycetia;o__Mycobacteriales;f__Mycobacteriaceae;g__Mycobacterium;s__Mycobacterium smegmatis |
| GCF_022370635.2 | 100 | 0 | 5965857 | 6183573 | 0.64 | | d__Bacteria;p__Actinobacteriota;c__Actinomycetia;o__Mycobacteriales;f__Mycobacteriaceae;g__Mycobacterium;s__Mycobacterium sp001886515 |
| GCF_022370755.2 | 99.99 | 0.68 | 6741306 | 6741306 | 0.67 | | d__Bacteria;p__Actinobacteriota;c__Actinomycetia;o__Mycobacteriales;f__Mycobacteriaceae;g__Mycobacterium;s__Mycobacterium goodii |
| GCF_022370835.2 | 100 | 0.11 | 3921563 | 3936223 | 0.69 | | d__Bacteria;p__Actinobacteriota;c__Actinomycetia;o__Mycobacteriales;f__Mycobacteriaceae;g__Mycobacterium;s__ |
| GCF_022374875.2 | 100 | 0.19 | 5750445 | 6107383 | 0.64 | | d__Bacteria;p__Actinobacteriota;c__Actinomycetia;o__Mycobacteriales;f__Mycobacteriaceae;g__Mycobacterium;s__Mycobacterium rufum_A |
| GCF_022374895.2 | 100 | 0 | 5901783 | 6163560 | 0.65 | | d__Bacteria;p__Actinobacteriota;c__Actinomycetia;o__Mycobacteriales;f__Mycobacteriaceae;g__Mycobacterium;s__Mycobacterium lentiflavum |
| GCF_022374935.2 | 100 | 0.21 | 4980142 | 4980142 | 0.67 | | d__Bacteria;p__Actinobacteriota;c__Actinomycetia;o__Mycobacteriales;f__Mycobacteriaceae;g__Mycobacterium;s__Mycobacterium heraklionense_A |
| GCF_022430545.2 | 100 | 0.43 | 5522624 | 5522624 | 0.66 | | d__Bacteria;p__Actinobacteriota;c__Actinomycetia;o__Mycobacteriales;f__Mycobacteriaceae;g__Mycobacterium;s__ |
| GCF_024320865.1 | 100 | 0.06 | 406159 | 6822461 | 0.68 | | d__Bacteria;p__Actinobacteriota;c__Actinomycetia;o__Mycobacteriales;f__Mycobacteriaceae;g__Mycobacterium;s__ |
| GCF_025215495.1 | 100 | 0.04 | 3988920 | 3988920 | 0.69 | | d__Bacteria;p__Actinobacteriota;c__Actinomycetia;o__Mycobacteriales;f__Mycobacteriaceae;g__Mycobacterium;s__Mycobacterium brumae |
| GCF_025331195.1 | 100 | 0.29 | 1047412 | 6577636 | 0.65 | | d__Bacteria;p__Actinobacteriota;c__Actinomycetia;o__Mycobacteriales;f__Mycobacteriaceae;g__Mycobacterium;s__Mycobacterium llatzerense |
| GCF_025345615.1 | 99.83 | 0.1 | 1023639 | 5632578 | 0.56 | | d__Bacteria;p__Actinobacteriota;c__Actinomycetia;o__Mycobacteriales;f__Mycobacteriaceae;g__Mycobacterium;s__ |
| GCF_025821625.1 | 100 | 0.49 | 64656 | 6688400 | 0.65 | | d__Bacteria;p__Actinobacteriota;c__Actinomycetia;o__Mycobacteriales;f__Mycobacteriaceae;g__Mycobacterium;s__ |
| GCF_025821985.1 | 100 | 0.06 | 170746 | 5931282 | 0.69 | | d__Bacteria;p__Actinobacteriota;c__Actinomycetia;o__Mycobacteriales;f__Mycobacteriaceae;g__Mycobacterium;s__Mycobacterium avium |
| GCF_025822065.1 | 100 | 0.13 | 165507 | 6885661 | 0.67 | | d__Bacteria;p__Actinobacteriota;c__Actinomycetia;o__Mycobacteriales;f__Mycobacteriaceae;g__Mycobacterium;s__Mycobacterium intermedium |
| GCF_025822245.1 | 99.85 | 0.47 | 185841 | 6529154 | 0.61 | | d__Bacteria;p__Actinobacteriota;c__Actinomycetia;o__Mycobacteriales;f__Mycobacteriaceae;g__Mycobacterium;s__ |
| GCF_025822405.1 | 100 | 0.48 | 437209 | 6673995 | 0.62 | | d__Bacteria;p__Actinobacteriota;c__Actinomycetia;o__Mycobacteriales;f__Mycobacteriaceae;g__Mycobacterium;s__Mycobacterium angelicum |
| GCF_025822555.1 | 100 | 0.03 | 1317408 | 6709978 | 0.65 | | d__Bacteria;p__Actinobacteriota;c__Actinomycetia;o__Mycobacteriales;f__Mycobacteriaceae;g__Mycobacterium;s__Mycobacterium fluoranthenivorans |
| GCF_025822605.1 | 100 | 3.32 | 330422 | 6920863 | 0.66 | | d__Bacteria;p__Actinobacteriota;c__Actinomycetia;o__Mycobacteriales;f__Mycobacteriaceae;g__Mycobacterium;s__ |
| GCF_025822705.1 | 99.98 | 0 | 232585 | 5973602 | 0.63 | | d__Bacteria;p__Actinobacteriota;c__Actinomycetia;o__Mycobacteriales;f__Mycobacteriaceae;g__Mycobacterium;s__Mycobacterium flavescens_A |
| GCF_025822765.1 | 99.99 | 0.01 | 310873 | 5900227 | 0.64 | | d__Bacteria;p__Actinobacteriota;c__Actinomycetia;o__Mycobacteriales;f__Mycobacteriaceae;g__Mycobacterium;s__ |
| GCF_025822865.1 | 100 | 0 | 428447 | 5402488 | 0.62 | | d__Bacteria;p__Actinobacteriota;c__Actinomycetia;o__Mycobacteriales;f__Mycobacteriaceae;g__Mycobacterium;s__Mycobacterium sp001665605 |
| GCF_025823185.1 | 100 | 0.87 | 608029 | 6452568 | 0.63 | | d__Bacteria;p__Actinobacteriota;c__Actinomycetia;o__Mycobacteriales;f__Mycobacteriaceae;g__Mycobacterium;s__ |
| GCF_025823315.1 | 100 | 0 | 942544 | 6461255 | 0.58 | | d__Bacteria;p__Actinobacteriota;c__Actinomycetia;o__Mycobacteriales;f__Mycobacteriaceae;g__Mycobacterium;s__Mycobacterium houstonense |
| GCF_026011875.1 | 100 | 0.41 | 126187 | 6484216 | 0.59 | | d__Bacteria;p__Actinobacteriota;c__Actinomycetia;o__Mycobacteriales;f__Mycobacteriaceae;g__Mycobacterium;s__ |
| GCF_026242035.1 | 99.93 | 0.2 | 147621 | 6879965 | 0.7 | | d__Bacteria;p__Actinobacteriota;c__Actinomycetia;o__Mycobacteriales;f__Mycobacteriaceae;g__Mycobacterium;s__ |
| GCF_027529605.1 | 99.91 | 0.23 | 319503 | 5875958 | 0.7 | | d__Bacteria;p__Actinobacteriota;c__Actinomycetia;o__Mycobacteriales;f__Mycobacteriaceae;g__Mycobacterium;s__ |
| GCF_030519285.1 | 99.83 | 0.13 | 20474 | 6212204 | 0.7 | | d__Bacteria;p__Actinobacteriota;c__Actinomycetia;o__Mycobacteriales;f__Mycobacteriaceae;g__Mycobacterium;s__ |
| GCF_034555895.2 | 100 | 0.04 | 5804834 | 6268691 | 0.65 | | d__Bacteria;p__Actinobacteriota;c__Actinomycetia;o__Mycobacteriales;f__Mycobacteriaceae;g__Mycobacterium;s__Mycobacterium europaeum |
| GCF_900078675.2 | 94.16 | 1.73 | 4995064 | 5929237 | 0.65 | | d__Bacteria;p__Actinobacteriota;c__Actinomycetia;o__Mycobacteriales;f__Mycobacteriaceae;g__Mycobacterium;s__Mycobacterium interjectum |
| GCF_900078685.2 | 99.92 | 2.14 | 3843338 | 4849907 | 0.68 | | d__Bacteria;p__Actinobacteriota;c__Actinomycetia;o__Mycobacteriales;f__Mycobacteriaceae;g__Mycobacterium;s__Mycobacterium icosiumassiliensis |
| GCF_900108565.1 | 100 | 0 | 5987931 | 5987931 | 0.68 | | d__Bacteria;p__Actinobacteriota;c__Actinomycetia;o__Mycobacteriales;f__Mycobacteriaceae;g__Mycobacterium;s__Mycobacterium rutilum |
| GCF_900157365.1 | 100 | 5.19 | 4451009 | 6248949 | 0.65 | | d__Bacteria;p__Actinobacteriota;c__Actinomycetia;o__Mycobacteriales;f__Mycobacteriaceae;g__Mycobacterium;s__Mycobacterium numidiamassiliense |
| GCF_900157375.1 | 100 | 1.22 | 6015470 | 6015470 | 0.67 | | d__Bacteria;p__Actinobacteriota;c__Actinomycetia;o__Mycobacteriales;f__Mycobacteriaceae;g__Mycobacterium;s__Mycobacterium rhizamassiliense |
| GCF_900157385.1 | 100 | 4.17 | 1348111 | 6029582 | 0.68 | | d__Bacteria;p__Actinobacteriota;c__Actinomycetia;o__Mycobacteriales;f__Mycobacteriaceae;g__Mycobacterium;s__Mycobacterium terramassiliense |
| GCF_900176255.2 | 99.99 | 1.56 | 1699181 | 6120821 | 0.66 | | d__Bacteria;p__Actinobacteriota;c__Actinomycetia;o__Mycobacteriales;f__Mycobacteriaceae;g__Mycobacterium;s__Mycobacterium ahvazicum |
| GCF_900187065.1 | 100 | 0.01 | 4950685 | 4950685 | 0.69 | | d__Bacteria;p__Actinobacteriota;c__Actinomycetia;o__Mycobacteriales;f__Mycobacteriaceae;g__Mycobacterium;s__Mycobacterium thermoresistibile |
| GCF_900232995.1 | 100 | 0.27 | 3450000 | 6192024 | 0.62 | | d__Bacteria;p__Actinobacteriota;c__Actinomycetia;o__Mycobacteriales;f__Mycobacteriaceae;g__Mycobacterium;s__Mycobacterium simulans |
| GCF_900289185.1 | 100 | 0.41 | 3566527 | 6977991 | 0.67 | | d__Bacteria;p__Actinobacteriota;c__Actinomycetia;o__Mycobacteriales;f__Mycobacteriaceae;g__Mycobacterium;s__Mycobacterium porcinum |
| GCF_900292015.1 | 100 | 0.53 | 5607630 | 5607630 | 0.65 | | d__Bacteria;p__Actinobacteriota;c__Actinomycetia;o__Mycobacteriales;f__Mycobacteriaceae;g__Mycobacterium;s__Mycobacterium basiliense |
| GCF_900417275.1 | 99.99 | 0.13 | 4829736 | 4842631 | 0.66 | | d__Bacteria;p__Actinobacteriota;c__Actinomycetia;o__Mycobacteriales;f__Mycobacteriaceae;g__Mycobacterium;s__Mycobacterium shimoidei |
| GCF_900453395.1 | 100 | 1.09 | 867103 | 5251936 | 0.65 | | d__Bacteria;p__Actinobacteriota;c__Actinomycetia;o__Mycobacteriales;f__Mycobacteriaceae;g__Mycobacterium;s__Mycobacterium xenopi |
| GCF_900453455.1 | 100 | 0 | 3573766 | 6021188 | 0.69 | | d__Bacteria;p__Actinobacteriota;c__Actinomycetia;o__Mycobacteriales;f__Mycobacteriaceae;g__Mycobacterium;s__Mycobacterium chubuense |
| GCF_900453675.1 | 100 | 0.15 | 5289247 | 5313907 | 0.69 | | d__Bacteria;p__Actinobacteriota;c__Actinomycetia;o__Mycobacteriales;f__Mycobacteriaceae;g__Mycobacterium;s__Mycobacterium phlei |
| GCF_900566055.1 | 100 | 0.64 | 35686 | 6187059 | 0.64 | | d__Bacteria;p__Actinobacteriota;c__Actinomycetia;o__Mycobacteriales;f__Mycobacteriaceae;g__Mycobacterium;s__Mycobacterium innocens |
| GCF_900566075.1 | 100 | 0.04 | 6308341 | 6425786 | 0.66 | | d__Bacteria;p__Actinobacteriota;c__Actinomycetia;o__Mycobacteriales;f__Mycobacteriaceae;g__Mycobacterium;s__Mycobacterium pseudokansasii |
| GCF_900566085.1 | 100 | 0.23 | 54408 | 6527895 | 0.64 | | d__Bacteria;p__Actinobacteriota;c__Actinomycetia;o__Mycobacteriales;f__Mycobacteriaceae;g__Mycobacterium;s__Mycobacterium attenuatum |
| GCF_900603025.1 | 100 | 0.42 | 5269097 | 5269097 | 0.69 | | d__Bacteria;p__Actinobacteriota;c__Actinomycetia;o__Mycobacteriales;f__Mycobacteriaceae;g__Mycobacterium;s__Mycobacterium hassiacum |
| GCF_900637195.1 | 100 | 0.11 | 6035584 | 6035584 | 0.68 | | d__Bacteria;p__Actinobacteriota;c__Actinomycetia;o__Mycobacteriales;f__Mycobacteriaceae;g__Mycobacterium;s__Mycobacterium aurum |
| GCF_900637205.1 | 100 | 0 | 5461769 | 5461769 | 0.69 | | d__Bacteria;p__Actinobacteriota;c__Actinomycetia;o__Mycobacteriales;f__Mycobacteriaceae;g__Mycobacterium;s__Mycobacterium chitae |
| GCF_902168065.1 | 92.41 | 2.53 | 55889 | 3238528 | 0.67 | | d__Bacteria;p__Actinobacteriota;c__Actinomycetia;o__Mycobacteriales;f__Mycobacteriaceae;g__Mycobacterium;s__Mycobacterium tilburgii |
| GCF_963853645.1 | 99.96 | 0.23 | 6155284 | 6635210 | 0.76 | | d__Bacteria;p__Actinobacteriota;c__Actinomycetia;o__Mycobacteriales;f__Mycobacteriaceae;g__Mycobacterium;s__Mycobacterium riyadhense |
| GCA_003402475.1 (MFM001) | 99.98 | 0.13 | 347840 | 5006351 | 0.67 | | d__Bacteria;p__Actinobacteriota;c__Actinomycetia;o__Mycobacteriales;f__Mycobacteriaceae;g__Mycobacterium;s__Mycobacterium sp003402475 |

| **Table S3**. Pairwise average nucleotide identity (ANI) and digital DNA–DNA hybridization (dDDH) values among the clinical isolates, *Mycobacterium* sp. strain MFM001, and closely related *Mycobacterium* species. | | | |
| --- | --- | --- | --- |
| Comparison | ANI (%) | dDDH (%) | dDDH 95% CI (%) |
| *M. celatum* vs *M. kyorinense* | 90.49 | 39.9 | 37.4–42.4 |
| *M. celatum* vs *M. branderi* | 90.86 | 40.3 | 37.8–42.8 |
| *M. celatum* vs *M.* sp. KO_240205 | 90.08 | 37.9 | 35.4–40.4 |
| *M. celatum* vs *M*. sp. KO_240413 | 90.10 | 37.9 | 35.4–40.4 |
| *M. celatum* vs *M.* sp. MFM001 | 90.13 | 38.0 | 35.5–40.5 |
| *M. kyorinense* vs *M. branderi* | 90.81 | 40.8 | 38.3–43.3 |
| *M. kyorinense* vs *M.* sp. KO_240205 | 90.63 | 39.7 | 37.2–42.2 |
| *M. kyorinense* vs *M.* sp. KO_240413 | 90.64 | 39.7 | 37.2–42.2 |
| *M. kyorinense* vs *M.* sp. MFM001 | 90.69 | 39.8 | 37.4–42.4 |
| *M. branderi* vs *M.* sp. KO_240205 | 90.24 | 38.2 | 35.7–40.7 |
| *M. branderi* vs *M.* sp. KO_240413 | 90.24 | 38.2 | 35.7–40.7 |
| *M. branderi* vs *M.* sp. MFM001 | 90.22 | 38.1 | 35.6–40.6 |
| *M.* sp. KO_240205 vs *M.* sp. KO_240413 | 100 | 100 | 100.0–100.0 |
| *M.* sp. KO_240205 vs *M.* sp. MFM001 | 99.98 | 99.8 | 99.7–99.9 |
| *M.* sp. KO_240413 vs *M.* sp. MFM001 | 99.98 | 99.8 | 99.7–99.9 |

| **Table S4.** Drug susceptibility of the two clinical isolates, determined by broth microdilution with BrothMIC SGM. | | |
| --- | --- | --- |
| Drug | KO_240205 | KO_240413 |
| CAM | 0.125 | 0.25 |
| AZM | 0.25 | 1 |
| MFLX | ≤0.25 | ≤0.25 |
| STFX | 0.25 | 0.25 |
| AMK | ≤8 | ≤8 |
| KM | 4 | 4 |
| MINO | ≤0.5 | 4 |
| DOXY | 2 | 4 |
| INH | >4 | >4 |
| LZD | 4 | 8 |
| EB | ≤0.5 | 1 |
| TH | ≤0.5 | 1 |
| RBT | 2 | 2 |
| RFP | >4 | >4 |
| Drug susceptibility results are expressed in μg/mL.  Abbreviations: CAM, clarithromycin; AZM, azithromycin; MFLX, moxifloxacin; STFX, sitafloxacin; AMK, amikacin; KM, kanamycin; MINO, minocycline; DOXY, doxycycline; INH, isoniazid; LZD, linezolid; EB, ethambutol; TH, ethionamide; RBT, rifabutin; RFP, rifampicin. | | |

**Supplementary figures**

**Figure S1.**

(A) Chest radiograph showing bilateral granular opacities predominantly in the lower lung fields. (B) Chest computed tomography showing peripheral granular opacities, bronchiectasis (open arrowheads: ▷), bronchial wall thickening (solid arrowheads: ▶), and mucus plugging (arrows: →) in both lower lobes.

A

B

**References for Online Supplement**

[1] Chaumeil PA, Mussig AJ, Hugenholtz P, Parks DH. GTDB-Tk v2: memory friendly classification with the genome taxonomy database. Bioinformatics. 2022 Nov 30;38(23):5315-5316.
[2] Chklovski A, Parks DH, Woodcroft BJ, Tyson GW. CheckM2: a rapid, scalable and accurate tool for assessing microbial genome quality using machine learning. Nat Methods. 2023 Aug;20(8):1203-1212. doi: 10.1038/s41592-023-01940-w. Epub 2023 Jul 27. Erratum in: Nat Methods. 2024 Apr;21(4):735.
[3] Tanizawa Y, Fujisawa T, Nakamura Y. DFAST: a flexible prokaryotic genome annotation pipeline for faster genome publication. Bioinformatics. 2018 Mar 15;34(6):1037-1039.
[4] Page AJ, Cummins CA, Hunt M, Wong VK, Reuter S, Holden MT, et al. Roary: rapid large-scale prokaryote pan genome analysis. Bioinformatics. 2015 Nov 15;31(22):3691-3.
[5] Minh BQ, Schmidt HA, Chernomor O, Schrempf D, Woodhams MD, von Haeseler A, et al. IQ-TREE 2: New Models and Efficient Methods for Phylogenetic Inference in the Genomic Era. Mol Biol Evol. 2020;37(5):1530–1534.
[6] Jain C, Rodriguez-R LM, Phillippy AM, Konstantinidis KT, Aluru S. High throughput ANI analysis of 90K prokaryotic genomes reveals clear species boundaries. Nat Commun. 2018 Nov 30;9(1):5114.
[7] Meier-Kolthoff JP, Sardà Carbasse J, Peinado-Olarte RL, Göker M. TYGS and LPSN: a database tandem for fast and reliable genome-based classification and nomenclature of prokaryotes. Nucleic Acids Res. 2022 Jan; 50(D1):D801–D807.
